# Biological properties of influenza A virus mutants with amino acid substitutions in the HA2 glycoprotein of the HA1/HA2 interaction region

**DOI:** 10.1099/jgv.0.001305

**Published:** 2019-07-22

**Authors:** L. Jakubcová, M. Vozárová, J. Hollý, K. Tomčíková, M. Fogelová, K. Polčicová, F. Kostolanský, E. Fodor, E. Varečková

**Affiliations:** ^1^ Institute of Virology, Biomedical Research Center, Slovak Academy of Sciences, Dúbravská cesta 9, 845 05 Bratislava, Slovakia; ^2^ Sir William Dunn School of Pathology, University of Oxford, Oxford, UK

**Keywords:** influenza A virus, mutant, fusion pH optimum, pH stability, virulence, infectivity, mouse lethal dose

## Abstract

Influenza A viruses (IAVs) enter into cells by receptor-dependent endocytosis. Subsequently, conformational changes of haemagglutinin are triggered by low environmental pH and the N terminus of HA2 glycoprotein (gp) is inserted into the endosomal membrane, resulting in fusion pore formation and genomic vRNA release into the cytoplasm. However, the pH optimum of membrane fusion is host- and virus-specific and can have an impact on virus pathogenicity. We prepared mutants of neurotropic IAV A/WSN/33 (H1N1) with aa substitutions in HA2 gp at the site of HA1/HA2 interaction, namely T64_2_H (HA2 numbering position 64, H1 numbering position HA407; referred to as mutant '64'), V66_2_H ('66') (HA409); and a double mutant ('D') with two aa substitutions (T64_2_H, V66_2_H). These substitutions were hypothesized to influence the pH optimum of fusion. The pH optimum of fusion activity was measured by a luciferase assay and biological properties of viruses were monitored. The *in vitro* and *in vivo* replication ability and pathogenicity of mutants were comparable (64) or lower (66, D) than those of the wild-type virus. However, the HA2 mutation V66_2_H and double mutation T64_2_H, V66_2_H shifted the fusion pH maximum to lower values (ranging from 5.1 to 5.3) compared to pH from 5.4 to 5.6 for the wild-type and 64 mutant. The decreased replication ability and pathogenicity of 66 and D mutants was accompanied by higher titres in late intervals post-infection in lungs, and viral RNA in brains compared to wild-type virus-infected mice. These results have implications for understanding the pathogenicity of influenza viruses.

## Introduction

Influenza A viruses (IAVs) are common respiratory pathogens, which under certain circumstances cause a serious disease with a fatal outcome. The broad host range and high variability of IAVs are linked to influenza epidemics and pandemics. To better understand the virulence potential of new influenza viruses in humans, the factors influencing the virulence of IAVs need to be studied [1, 2]. The surface glycoprotein (gp) haemagglutinin (HA) plays a major role in virulence [3–5]. HA forms a trimer composed of three identical monomers. HA molecules are proteolytically cleaved into HA1 and HA2 glycopolypeptides intra- or extracellularly, depending on the structure of the cleavage site. The HA1 gp (heavy chain) represents a receptor binding subunit and the HA2 gp (light chain) is a fusion subunit. HA plays an important role during the first steps of IAV replication in permissive cells. It is responsible for determining the host range of IAVs, as it binds to sialic acid receptors linked to glycoproteins or glycolipids on the surface of target cells in a host-specific manner. The HA derived from IAVs of various subtypes and host origin is able to differentiate cell receptors according to their structure, thus determining the first step of IAV infection. The second important role of HA lies in its ability to mediate the fusion of the virus with endosomal membranes after IAV internalization into the cell.

The fusion process mediated by IAV is activated by the low pH environment in endosomes, where HA undergoes large irreversible structural changes [6]. The pH needed to trigger the fusion process is dependent on the HA structure and ranges from pH 5 to 6 [7]. The refolding of HA in an acidic environment, which precedes fusion pore formation, starts by weakening of intermolecular bonds in the HA trimer, leading to an increase in the distance between globular domains of HA monomers. Consequently, HA2 gp that in native conformation is covered by the HA1 globular part is exposed and its N terminus is inserted into the endosomal membrane. Finally, the fusion pore is formed, enabling the release of viral genome into the cytoplasm and its transport to the site of vRNA replication [8]. Although many studies have described the mechanism of IAV and endosomal membrane fusion, the details of this process are not yet clear [9, 10].

The fusion of viral and cell membranes is essential for the infectivity of IAV and its replication in the host [4, 5]. Understanding the factors and molecular requirements that lead to the efficient growth of virus in cell culture or in animal models is of fundamental importance for viral diseases such as influenza. The pH optimum of IAV and endosomal membrane fusion is host- and virus-specific and can have an impact on virus pathogenicity as well as on the ability of the virus to replicate at the site of virus entry in a given host [5, 11–13]. In this study we address the relationship between the pH optimum of virus–host membrane fusion and the virulence of IAV. By examining the known three-dimensional structure of various HAs available from the Protein Data Bank (http://www.rcsb.org) we designed and introduced amino acid substitutions at the interface of HA2 gp and HA1 gp of A/WSN/33 (H1N1) virus. By reverse genetics were prepared three viruses with mutations in HA2 gp: T64_2_H (HA2 numbering as from the N terminus of HA2 gp after HA0 cleavage, i.e. H1 numbering position HA407), V66_2_H (numbering as for HA2, i.e. H1 numbering position HA409) and a double mutant with both amino acid substitutions in HA2 (T64_2_H and V66_2_H). Molecular interactions at the HA1 and HA2 interface determine the pH required for fusion activity of IAV, and thus amino acid substitutions observed or introduced in this region may have further implications for virus virulence and pathogenicity. We examined the impact of the introduced mutations on the pH stability of mutant viruses and their replication ability *in vitro* and *in vivo* as well as their pathogenicity in a mouse model.

## Methods

### Viruses

Recombinant wild-type (wt) and mutant influenza A/WSN/33 (H1N1) viruses were generated by reverse genetics using the pHW2000 eight-plasmid system [14]. Mutations were introduced into the plasmid pHW2000 HA by site-directed mutagenesis to generate mutant viruses 64 (T64_2_H substitution in the HA2 subunit; H1 numbering – position 407 in HA); 66 (V66_2_H substitution in the HA2 subunit; H1 numbering – position 409 in HA) and D (T64_2_H and V66_2_H substitutions in the HA2 subunit). Viruses were propagated in the allantoic fluid of 9-day-old fertilized chicken eggs.

### Cells

Madin-Darby Canine Kidney (MDCK) cells, Madin-Darby Bovine Kidney (MDBK) epithelial cells, adenocarcinoma alveolar basal epithelial cells A549, human embryonic kidney 293T (HEK 293T) cells, and VERO African green monkey kidney cells were propagated in Dulbecco’s modified Eagle’s medium (DMEM; Lonza) supplemented with 10 % FBS (Lonza), 40 μg ml^−1^ gentamicin (Sandoz) and 2 mM glutamine (Lonza). BSRT-7/5 (BHK-21 – Baby Hamster Kidney cell line expressing T7 RNA polymerase) cells were propagated in the same medium supplemented with geneticin (1 mg ml^−1^) instead of gentamicin.

### Construction of IAVs by reverse genetics

The wild-type A/WSN/33 (H1N1) virus, as well as its HA2 mutants, were prepared by transfection of HEK 293T cells with eight reverse genetics plasmids bearing the genetic information corresponding to individual gene segments encoding all IAV proteins using Lipofectamine 2000 (Invitrogen) as the transfection reagent [14]. Transfected cells were incubated in Opti-MEM medium (Gibco) for 24 h at 37 °C. The medium was then replaced with MEM (minimum essential medium) containing 0.5 % FBS. Medium containing rescued virus was harvested 72 h after transfection, cleared by centrifugation (2000 r.p.m./5 min) and used for infection of MDBK cell monolayers. The presence of virus propagation in MDBK cells was monitored by light microscopy. When a cytopathic effect (as an indicator of virus replication) was visible on the cell monolayer, the culture medium containing the virus was harvested (from 24 to 48 h after infection) and infectious medium was aliquoted and stored at −80 °C.

### Acid stability determination of viruses by plaque assay

The virus inactivation test at low pH was performed using the method described by Zaraket *et al*. [12] with modifications. Briefly, aliquots of viruses (each 1×10^4^ p.f.u. ml^−1^) were treated for 5 min at 37 °C with 0.1 M MES buffer solution containing MES and its sodium salt, MES Na, of different pH ranging from pH 4.8 to pH 6.8 (ΔpH=0.2). After treatment, the pH of the virus suspension was neutralized with 1 M Tris (pH 8.6). Serial ten-fold-dilutions of treated virus suspensions were applied to MDCK cell monolayers in a 24-well plate (0.3 ml per well). Virus suspension was removed after 1 h of adsorption of the virus to the cell monolayer at room temperature and overlay medium [MEM containing 0.8 % agarose (AGTC Bioproducts), 0.5 % FBS, 2× non-essential amino acids (NEAA; Gibco), 4 mM ultraglutamine and 40 μg ml^−1^ gentamicin] was added. Plates were incubated at 37 °C in a humidified atmosphere containing 5 % CO_2_. After 72 h, infected cell monolayers were fixed with 20 % trichloroacetic acid, overlay medium was removed, and cells were washed with PBS and subsequently permeabilized by fixation with cold methanol at 4 °C for 20 min. After this interval mAb specific for IAV nucleoprotein 107L [15], at a concentration of 1.5 μg ml^−1^ in PBS containing 1 % bovine serum albumin was added. The bound mAb was detected with goat anti-mouse IgG conjugated with horseradish peroxidase (GAM IgG-Px; Bio-Rad) and the reaction was visualized by the addition of a substrate solution containing 3-amino-9-ethylcarbazole (Sigma) in 0.03 % H_2_O_2_. The visible immune-stained viral plaques were evaluated to determine infectious virus titre [16]. The experiment was repeated twice in quadruplicate for each virus. The virus titres were normalized, plotted and 50 % infectivity reduction was calculated using Graphpad Prism 7. The pH values at 50 % virus infectivity reduction were compared using ordinary one-way ANOVA.

### Cell–cell fusion luciferase reporter assay

A cell–cell fusion luciferase reporter assay was used to quantify the fusion activity of viruses at different pH. The assay was performed according to Galloway *et al*. [7] modified to determine pH optimum of membrane fusion in the context of infectious virus. VERO cells in six-well plates (cultured for 24 h in complete DMEM medium with 10 % FBS) were transfected with a plasmid expressing firefly luciferase under control of the T7 bacteriophage promoter using Turbofect (Thermo Scientific) transfection reagent. After 24 h, transfected cells were infected with wt virus, its mutants at m.o.i. 1 or mock infected as control. Untransfected VERO cells infected with wt virus were also included as a control. Twelve hours after infection, VERO cell monolayers were overlaid with BSRT-7/5 cells (10^6^ cells per well), constitutively expressing the bacteriophage T7 RNA polymerase and incubated for 1 h at 37 °C in DMEM without FBS. After this interval, medium was removed and infected cells were exposed to 0.1 M MES buffer (1 ml per well) adjusted to the appropriate pH ranging from pH 4.6 to 6 for 10 min at 37 °C. MES buffer was then replaced with the complete growth medium DMEM for 6 h to allow the IAV HA to mediate the fusion of infected VERO cells with target BSRT-7/5 cells, enabling the transfer of T7-luciferase plasmid into target cells and expression of firefly luciferase. Cells were lysed with 0.5 ml Reporter Lysis Buffer (Promega) and cell debris was removed by centrifugation at 15 000 ***g*** at 4 °C. Luciferase activity was measured in the supernatants using luciferase substrate oxyluciferin (Promega) and measured on a BioTek Synergy 2 Luminometer. Cell–cell fusion luciferase reporter assays were independently repeated at least three times in triplicate. Mock infected controls and no-transfection controls were done for boundary pH values (4.6 and 6.0) to determine the background signal. Data were normalized and analysed by non-linear regression fitting the bell-shaped curve using the least squares fit.

### Virus growth kinetics

Multistep growth curves in MDBK, A549 and VERO cells were determined for each recombinant virus. Confluent monolayers in six-well plates in triplicate were infected with an m.o.i. of 0.001 of each virus for 1 h at 37 °C. After this interval monolayers were washed and incubated at 37 °C for 12, 24, 36, 48, 60 and 72 h in MEM with 0.5 % FBS. The infectious virus titres in the supernatant of infected cells at each time-point were measured by a plaque assay [16–18].

### Animal experiments

Six-week-old female BALB/c mice provided by the Faculty of Medicine, Masaryk University, Brno, Czech Republic, were used. In all experiments presented in this study, the animals were treated according to the approval and standards of the European Union and State Veterinary and Food Administration of the Slovak Republic. The State Veterinary and Food Administration of the Slovak Republic specifically approved this study (permission number 1301/14–221). Fundamental ethical principles and animal welfare requirements were respected. For determination of 1LD_50_, groups of mice (*n*=5 per group) were intranasally (i.n.) infected with four different doses of examined virus diluted in PBS in a volume of 40 µl per mouse. Mice were anaesthetized with 2.5 % isoflurane in oxygen before virus inoculation. The survival of mice was monitored for 15 days. The infectious dose 1LD_50_ was determined by the method of Reed and Muench [19].

### Rapid culture assay (RCA)

To measure infectious virus in the lungs of infected mice, the lungs from three mice per group were excised at 2-day intervals. Lung homogenate suspensions in PBS (20 %, w/v) were prepared and cell debris was pelleted. The supernatants were examined for infectious virus as described [16, 20]. Briefly, MDCK cells were cultured in DMEM medium containing 5 % FBS in 96-well plates for 24 h to a density of 5×10^4^ cells per well. After removing the culture medium, two-fold dilutions of virus sample in PBS or serum-free DMEM (starting from 50×) were applied onto the cell monolayer for 1 h at 37 °C. Medium with un-adsorbed virus was then removed and Ultra-MDCK medium with 0.5 % FBS was added. After 18 h of incubation under the same conditions, cell monolayers were washed, fixed with cold methanol and the virus was detected using monoclonal antibody (mAb) 107L specific to IAV nucleoprotein as described before [15] (1.5 µg ml^−1^ in PBS with 5 % non-fat dry milk). After 1.5 h of incubation with HRP-conjugated goat anti-mouse IgG (1 : 1500; Dako), a positive reaction was visualized by addition of substrate solution containing 3-amino 9-ethyl-carbazole with 0.03 % H_2_O_2_. Stained red coloured cells identified by light microscopy were considered as positive. The virus titre was expressed as the reciprocal value of the highest sample dilution positive for replicating virus in cell culture [16].

### Quantitative real-time PCR

Quantitative real-time PCR was performed on a StepOne Real-Time PCR System (Applied Biosystems) using Maxima SYBR Green/ROX qPCR Master Mix (Thermo Fisher Scientific) according to the manufacturer’s instructions. To detect vRNA, a volume of 150 µl of supernatant from brain cell homogenates was used. Total RNA was extracted by the phenol–chloroform method and examined for vRNA [21]. Total RNA from brain cell homogenates was transcribed to cDNA using random hexamer primers (Thermo Fisher Scientific) and M-MuLV reverse transcriptase (Fermentas). The primers specific for IAV NS1 (FW: 5′-GATTCGCTTGGAGAAGCAGT-3′; REV: 5′-AAATAAGCTGAAACGAGAAAGTTC-3′) and β-actin (FW: 5′-CCAACCGCGAGAAGATGACC-3′; REV: 5′-GATCTTCATGAGGTAGTCAGT −3′) were used for the reaction. The amplification cycle was as follows: 10 min at 95 °C, 40 cycles of 15 s at 95 °C and 1 min at 60 °C. The relative amount of NS1 RNA was calculated using the ΔΔCT method. The expression level of NS1 was normalized to β-actin. To determine the change in NS1 expression, the normalized expression of NS1 in each sample was divided by the normalized expression of NS1 in the control sample, which was arbitrarily set to be the wt-infected mouse B at 2 days post-infection. We use vRNA as a general term to refer to all types of viral RNA, including genomic negative sense vRNA, anti-genomic positive-sense cRNA and mRNA.

## Results

### Design and rescue of HA2 mutants

**Fig. 1.**
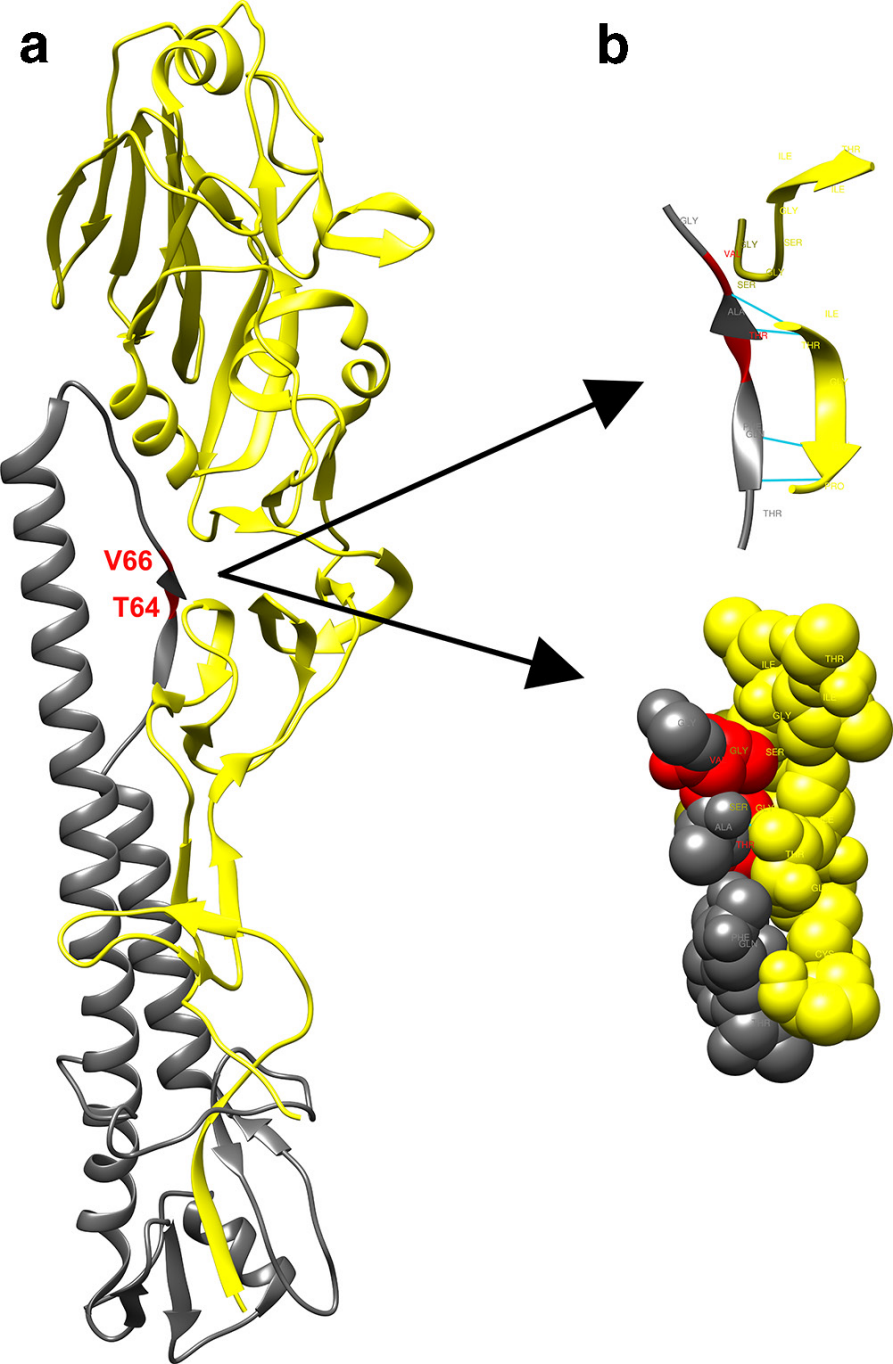
The structure of HA monomer as determined by X-ray crystallography. The figure was created using UCSF Chimera (http://www.rbvi.ucsf.edu/chimera). (a) HA monomer with red labelled substitutions in HA2, HA1 gp is coloured yellow and HA2 gp grey. (b) Substituted amino acids in the light chain of HA (HA2 gp: grey) on the molecular level (T64 a V66: red) and its interaction with the heavy chain of HA (HA1 gp: yellow). Hydrogen bonds: cyan.

To assess the influence of amino acids at critical positions of the HA trimer on the optimal pH value of IAV fusion, we prepared mutant viruses with amino acid substitutions at the interface between the globular and stem parts of the HA trimer. The positions for introduced mutations were selected on the basis of the known 3D structure of the HA trimer (Fig. 1). The interaction at the interface of the HA1 globular part and the HA2 stem is mediated by hydrogen bonds and weak interactions, which are influenced by the environmental pH. From this point of view histidine is considered as a critical amino acid for pH-dependent changes in the structure of proteins [22]. The conjugate acid (protonated form) of the imidazole side chain in histidine has a pKa of approximately 6.0. Histidine was therefore introduced into the light chain of HA in two positions, namely at position 64 instead of threonine (T64_2_H, referred to as mutant 64) and at position 66 instead of valine (V66_2_H, referred to as mutant 66). The third mutant we prepared contained both substitutions, i.e. T64_2_H and V66_2_H (this double mutant is referred to as ‘D’).

Amino acid substitutions were introduced by site-directed mutagenesis into the HA gene of IAV of H1N1 subtype A/WSN/33 (H1N1) and mutant viruses were generated using reverse genetics. The presence of the HA mutations was confirmed in the rescued viruses after their propagation in cells and fertilized chicken eggs by Sanger sequencing of the HA gene. Haemagglutinin and neuraminidase (NA) sequences of the rescued recombinant viruses were confirmed to have no other mutations for three consecutive passages in embryonated chicken eggs (data not shown).

### Determination of pH optimum of fusion activity of the HA2 mutant viruses

**Fig. 2.**
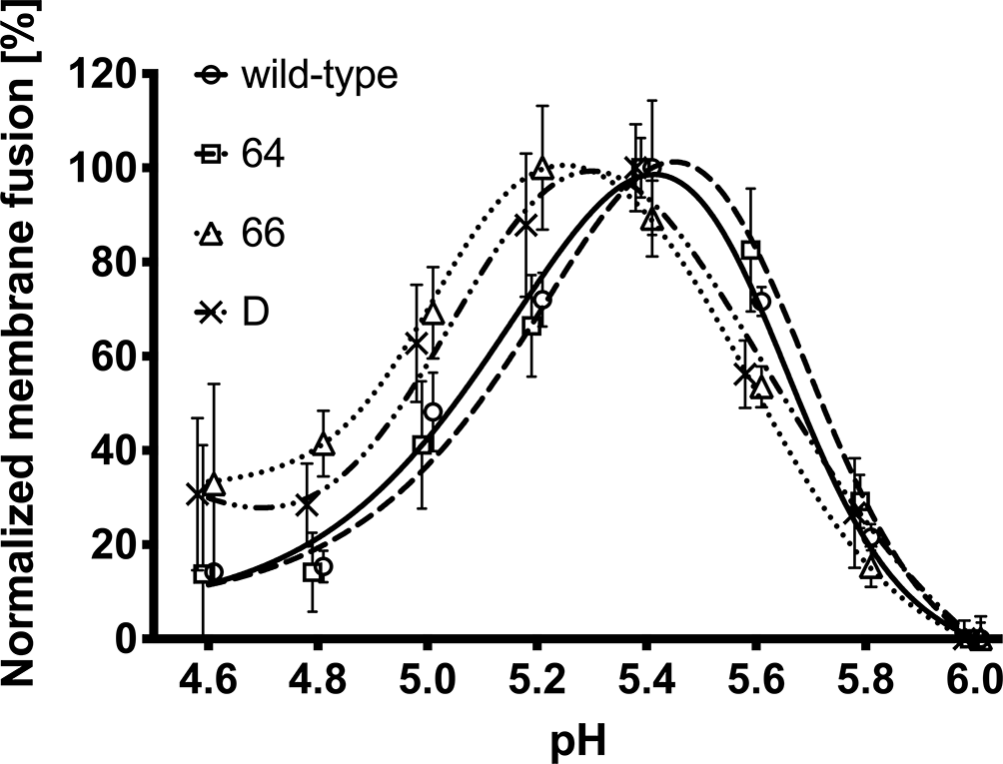
Determination of the pH optimum of membrane fusion mediated by constructed viruses using a luciferase reporter assay. VERO cells were transfected with plasmid expressing firefly luciferase under control of the T7 bacteriophage promoter. After transfection, cells were infected with influenza virus A/WSN/1933(H1N1) or its mutants. Then the infected, HA-expressing cells were overlaid with BSR-T7/5 cells, which constitutively express the bacteriophage T7 RNA polymerase. BSR-T7/5 cells were allowed to adhere and the pH of medium was then adjusted with MES buffer to different examined values. After incubation the MES buffer was replaced with complete growth medium and incubated at 37 °C for 6 h. The luciferase activity resulting from the fused cell populations was quantified by measuring luminescence. Non-infected VERO cells were used as a mock control for evaluation of the experimental data. Data points represent normalized means from three independent experiments, and error bars represent standard deviations. The points were nudged by +/–0.01 units along the *x* axis to allow the error bars be distinguished. The fusion pH was evaluated as described in the Methods section.

The pH optimum of fusion and biological properties of the IAV mutants were compared with the parental wt virus *in vitro* and *in vivo*. To monitor viral and endosomal membrane fusion we used a modified luciferase fusion assay described by Galloway *et al*. [7], which enabled quantification of the fusion process. The principle of this method is that the HA expressed on the surface of VERO cells is exposed to low pH, undergoes a conformational change into the fusion active form and mediates the creation of fusion pores between the plasma membranes of VERO cells and membranes of overlaid BSR-T7/5 cells. After the fusion of VERO and BSR-T7/5 cell membranes, the production of luciferase is triggered and measured (for details see Methods). The level of luminescence represents the rate of HA fusion activity. It has to be stressed here that we used infectious viruses to evaluate the fusion activity of HA and not HA-transfected cells or cells stably expressing HA. Therefore, our experimental system evaluates membrane fusion in the context of virus with most of its components to mimic the processes taking place in the endosome during virus entry and membrane fusion. We compared the pH of maximal measured fusion activity, namely the pH optimum of fusion, of all three mutants with that of wt virus in the pH range of 4.6–6.0 (Δ 0.2) (Fig. 2). No changes in relative luminiscence below pH 4.6 and above pH 6 were recorded. Results were obtained from three independent experiments in which all viruses were tested in triplicate. The high complexity of the assay in the context of virus infection led to high inter- and intra-assay variability. The pH optimum of membrane fusion for mutants 66 and D was in the range 5.1–5.3, and shifted to the lower values when compared to the wt and 64 virus pH optimum of membrane fusion within the interval 5.4–5.6 (Fig. 2). The exact pH optimum could not be determined due to the broad pH maxima evaluated (or ambiguous results) and rather intervals of pH optima of membrane fusion and shift to lower or higher values were taken into account.

### Acid stability of HA2 mutant viruses

**Fig. 3.**
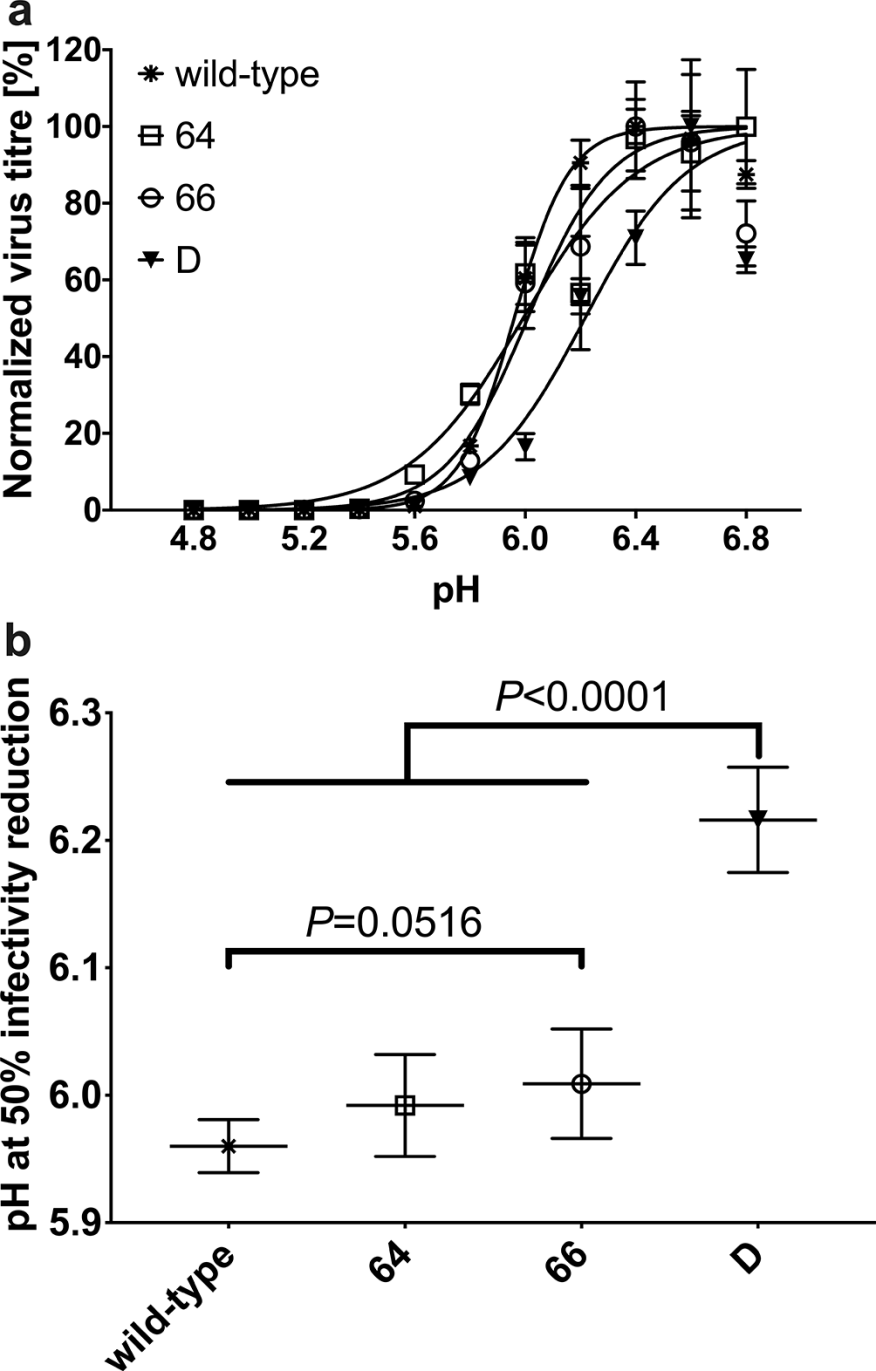
Infectious virus titre of wt and mutant viruses exposed to different pH environment. Viruses were exposed to a different pH (4.8–6.8; Δ0.2), then neutralized and virus titre was determined by plaque immunostaining. (a) Titres of infectious viruses obtained after their exposure to each pH were normalized and plotted against the corresponding pH values. Error bars represent standard deviations (*n*=4). (b) pH values at which the infectivity of viruses was reduced by 50 % were determined (IR50^pH^) and plotted. Standard errors and *P*-values are shown.

It is known that pre-exposure of virus to the critical low pH before infection causes irreversible conformational change of HA, which results in the loss of virus infectivity. The activation pH triggering such refolding of HA is characteristic to the particular virus strain. We evaluated the acid stability of the HA2 mutant viruses after their exposure to different pH, ranging from 4.8 to 6.8 (Δ 0.2) for 10 min at 37 °C. The infectivity of all viruses was determined by a modified plaque assay on MDCK cells [16]. Compared with the low pH stability of the wt and mutant viruses, we observed that the wt and mutants retained the highest infectivity at pH 6.4–6.8 (Fig. 3a), which decreased after their exposure to a low pH environment. At pH 5.8 their infectivity was reduced to less than 30 % of the original value (Fig. 3a). We determined the pH value causing 50 % reduction of virus infectivity (IR50^pH^) for each virus (Fig. 3b). We observed significant difference between the double mutant virus and other viruses. Double mutant virus was significantly more sensitive to low pH treatment (IR50^pH^=6.2) than other tested mutants (IR50^pH^=6.00) and the wt virus (IR50^pH^=5.96).

### *In vitro* replication kinetics of HA2 mutant viruses

**Fig. 4.**
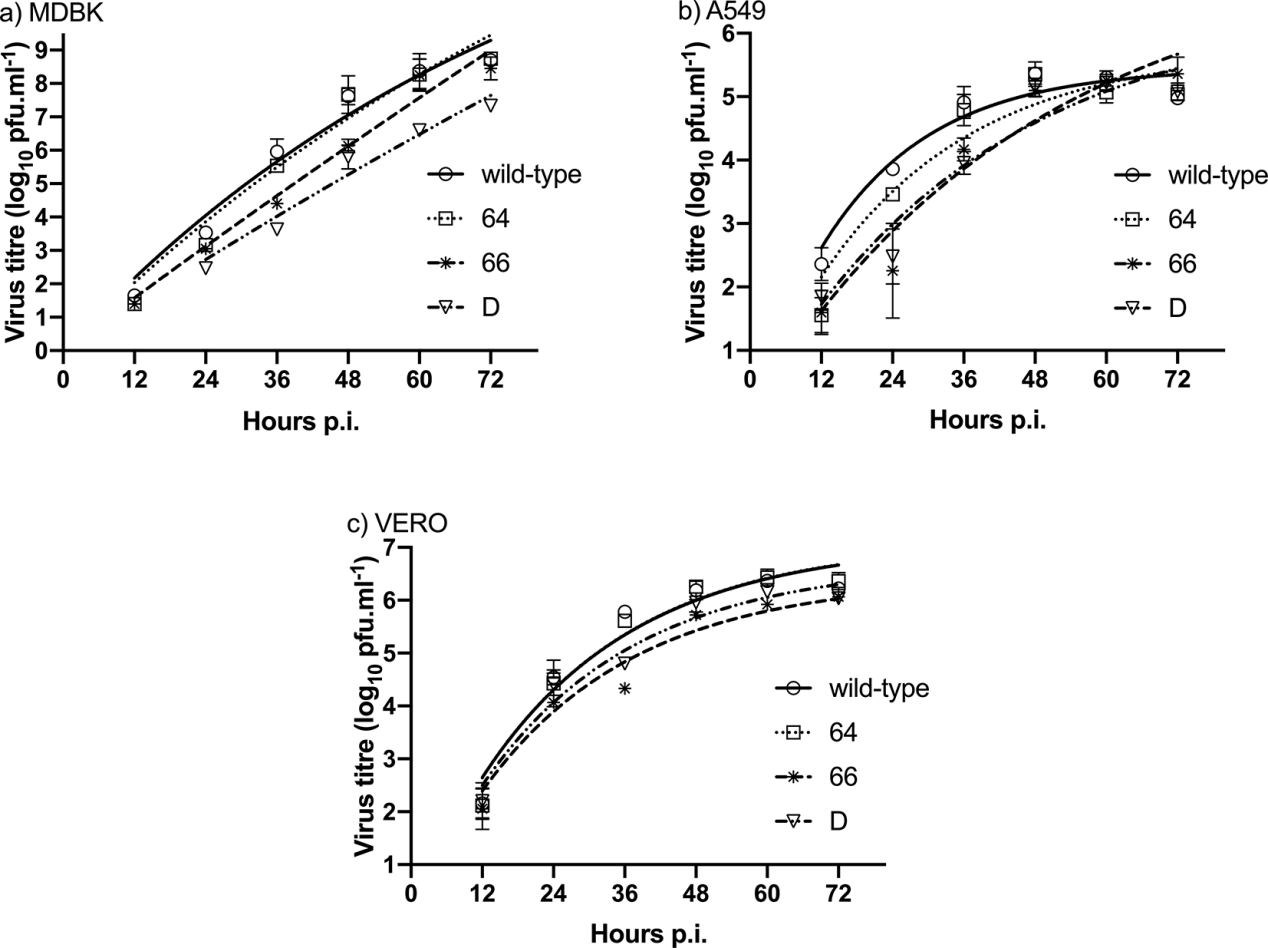
Viral growth on different cell lines: MDBK (a), A549 (b) or VERO (c) cell monolayers were inoculated with constructed viruses at an equal m.o.i. (0.001). Cell culture supernatants were collected from infected cell monolayers every 12 h and the viral titres were evaluated by an immunostaining plaque assay at each given time-point. Error bars represent the standard deviations from four parallel measurements.

To determine the replication properties of the viruses with mutations in the HA2 gp, we measured the multicycle replication of recombinant viruses (m.o.i. 0.001) in MDBK, A549 and VERO cells (Fig. 4). All recombinant viruses replicated robustly particularly in MDBK cells, with maximal titres close to 1×10^9^ p.f.u. ml^−1^. Maximal viral titres of all viruses were approximately 2–4 logs lower in A549 cells and 2–3 logs lower in VERO cells. The differences were observed in the exponential phase of virus growth in each cell line, namely 66 and D viruses reached lower titres than the wt and 64 mutant. However, at later time-points, the titre of examined viruses in a given cell line reached a plateau, the height of which was dependent on the cell type: it was reached after 48 h in A549 cells, where the maximal titre of all viruses was lower (5.1–5.35 log_10_) than in the other two cell lines. The maximal titre in VERO cells after 60 h was about 1 log_10_ higher than in A549 cells. In MDBK cells, a plateau was reached after the 72 h for wt, 64 and 66 viruses (titre 8.5 log_10_), where the log_10_ of the maximal titre was 8.4–8.5 and was higher than that of the double mutant virus (D) (log_10_ of the maximal titre was 7.3). However, the *in vitro* replication profile of mutant 64 was close to that of the wt virus in all three cell lines (Fig. 4). No significant differences in virus growth rates were observed in MDBK and VERO cells. In the A549 cell line, growth rates of 66 and D virus mutants were lower (*P*<0.01) than wt virus.

### Pathogenic properties of HA2 mutant viruses

To compare the pathogenicity of each virus mutant with wt virus, we determined the amount of pfu causing lethal infection of mice, corresponding to 1 LD_50_. Mice (*n*=5 per dose per virus) were infected with five different doses of a given virus and the course of the infection as well as survival of mice was monitored (results not shown). The *in vivo* titration of viruses showed that a similar infectious dose of mutant virus 64 (1.3×10^3^ p.f.u. 40 µl^–1^ per mouse) and wt virus (1.4×10^3^ p.f.u. 40 µl^–1^ per mouse) was required to achieve 50 % lethality for mice (1 LD_50_). The infectious dose required for the 50 % lethality of mice was 30 times higher for mutant 66, i.e. 4.0×10^4^ p.f.u. 40 µl^–1^ and seven times higher for the double mutant, i.e. 1.0×10^4^ p.f.u. 40 µl^–1^ in comparison to the wt virus. Thus mutant 66 and double mutant D were less virulent (pathogenic) than wt and 64 viruses.

### *In vivo* replication kinetics of HA2 mutant viruses

#### The presence of infectious virus in lungs of infected mice

**Fig. 5.**
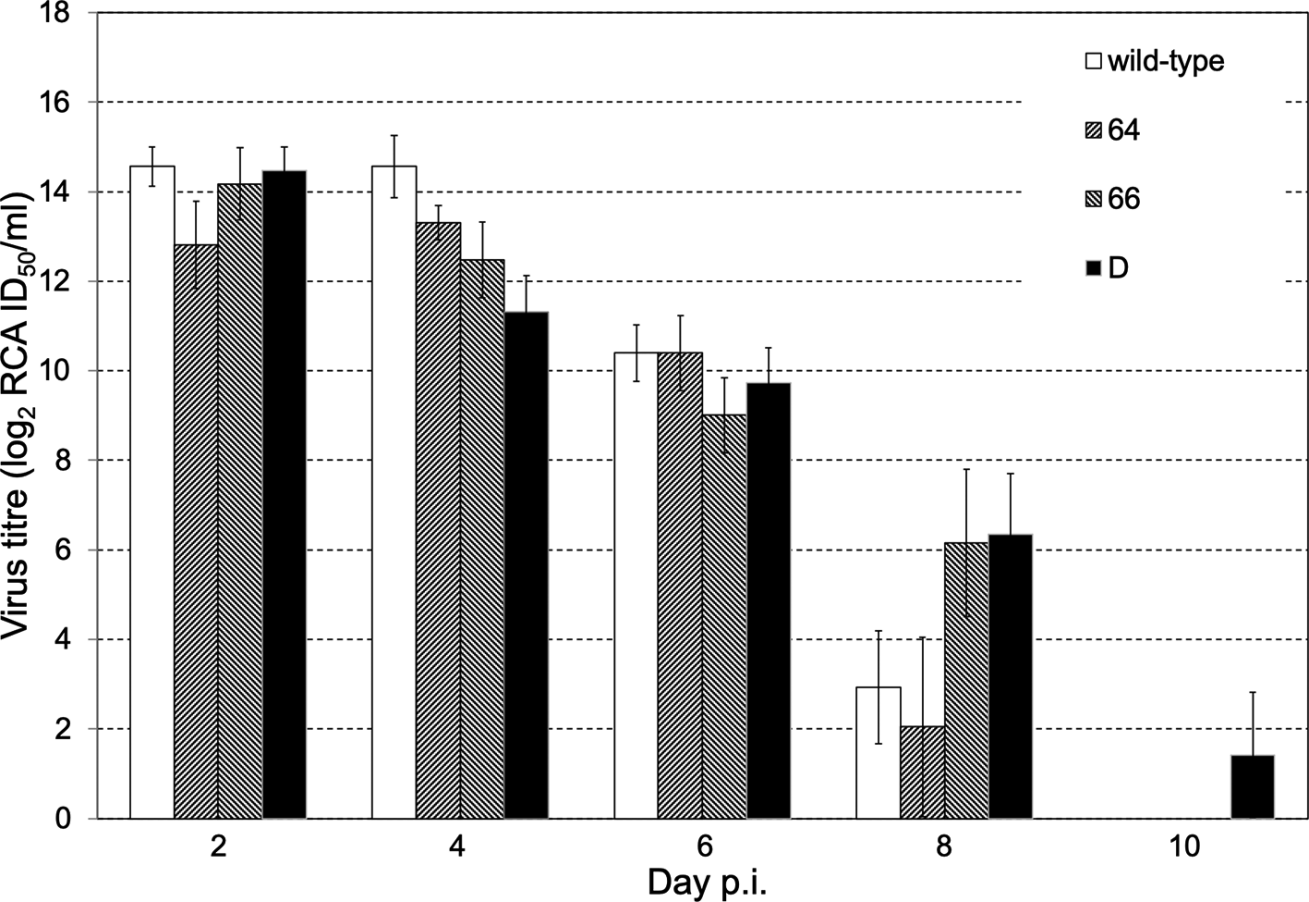
Kinetics of replication of examined viruses in lungs of mice infected with 1 LD_50_ of particular viruses. Virus titres in lungs of infected mice were determined by the RCA method on MDCK cells. Values on the *y* axis represent the mean value of viral titres (log_2_) obtained from three experimental individuals for each virus and each time-point. Error bars represent the standard deviations from four parallel measurements.

We compared the course of disease in HA2 mutant viruses’ infected mice (*n*=15 per virus) and the virus replication in mouse lungs (*n*=3 mice per interval per virus). To normalize the impact of infection, we used an infectious dose of 1 LD_50_ of each virus. The virus titre in lungs of mice infected with 1 LD_50_ was determined by rapid culture assay (RCA). All viruses reached maximal replication on day 2 or 4 post-infection, with peak viral titres ranging from 13.31 (log_2_) for virus 64 to 14.56 (log_2_) for wt virus. Interestingly, mutant viruses 66 and D remained in mouse lungs longer, at late points post-infection (8 days p.i.), and reached higher titres than wt and 64 (Fig. 5). The mutant virus D was present in lungs even 10 days after infection. These observations were consistent with the detection of vRNA in lungs by RT-PCR (results not shown).

#### Pathologic changes in lungs of infected mice

Simultaneously we examined the lung damage in mice infected with a dose 1 LD_50_ of each HA2 mutant virus in different time-points after the infection (Table 1). Pathological changes (lesions and oedema) macroscopically visible in lungs of mice (*n*=3 per group per interval) infected with wt and 64 viruses were comparable, being most severe on days 8–10 post-infection. The damage to lungs of mice infected with mutant virus 66 was not as severe and lungs were not oedematous, suggesting a milder course of infection caused by the 66 mutant. Although the lung damage of virus D-infected mice was milder than that of the wt and 64-infected mice, it was still higher than in 66-infected mice. Moreover, we were able to detect vRNA in lungs of mice infected with mutant D for a longer period of time, even on day 15 p.i. (two of three mice) (data not shown).

**Table 1.** Macroscopic lung damage in mice infected with 1 LD_50_ of examined viruses

Virus 1LD_50_	Lung damage in infected mice (%) Day p.i.
2	4	6	8	10
**Wild-type** 1.4×10^3^ p.f.u.	<5	10–20	70–90	70–90	70–90
**64** 1.3×10^3^ p.f.u.	0	10–20	50–70	70–90	70–90
**66** 4.0×10^4^ p.f.u.	0	<10	10–20	10–20	10–20
**D** 1.0×10^4^ p.f.u.	0	5–10	50–70	50–70	50–70

0 %, no pathological changes observed; 5–90 %, the extent of lung damage macroscopically visible (lesions and oedema). The pathological changes in lungs of mice infected with the particular viruses were examined in three mice per each time-point for each virus. Values represent the averages of observed lesions in lungs of infected mice expressed as the percentage lung damage, i.e. from three mice per group per time-interval.

#### The presence of infectious virus and vRNA in brains of infected mice

**Fig. 6.**
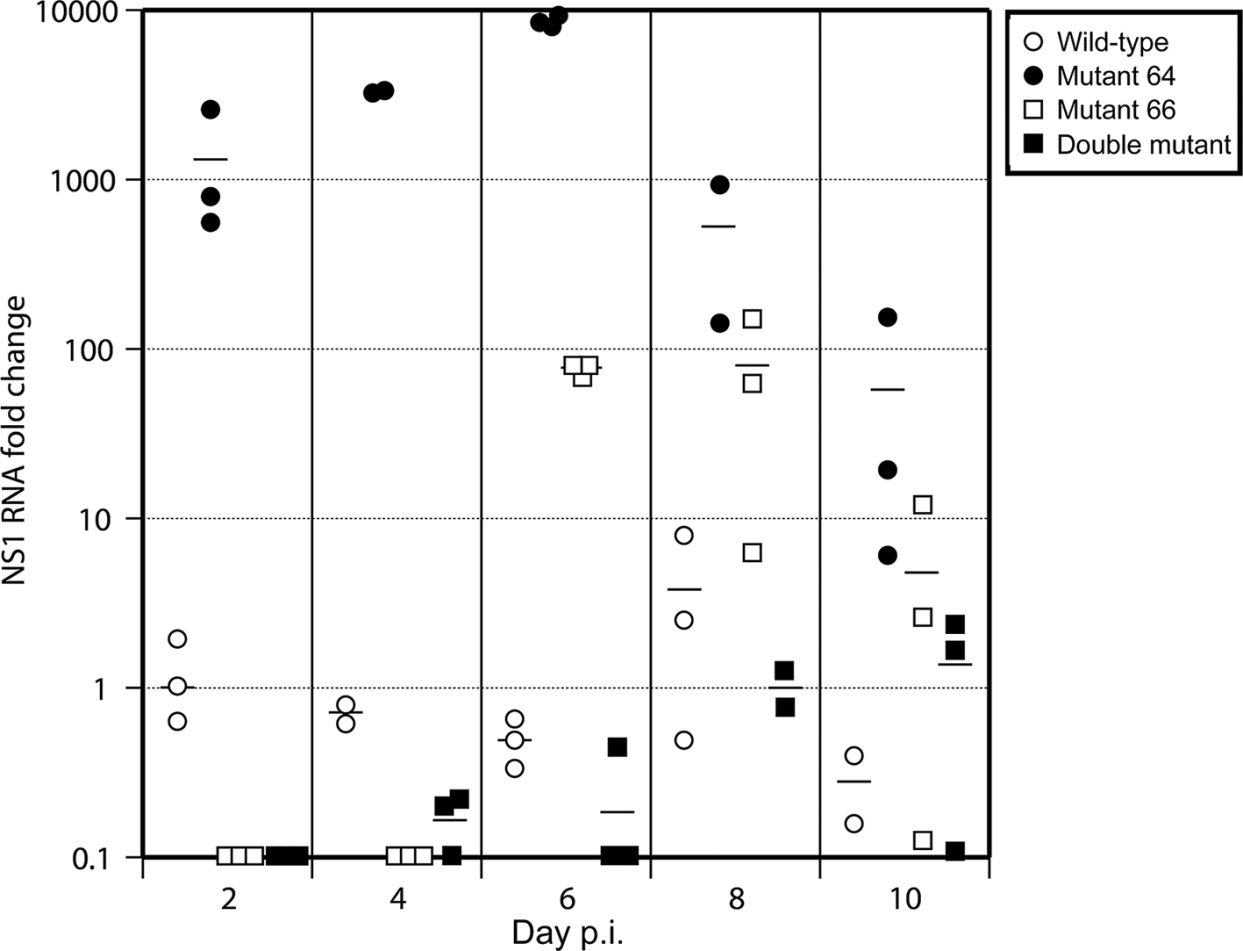
Relative amount of NS1 RNA in brains of infected mice. The expression level of vRNA of NS1 was determined using NS1-specific primers (see Methods). Quantitative real-time PCR was performed with samples taken at designated intervals after the infection of mice (*n*=3 per group per time point) and normalized to the wild type-infected mouse B at 2 days p.i. Each experimental point represents the average (bars) from three parallel measurements (points). Samples were collected in each interval from three mice per group and used individually for analysis.

Influenza A/WSN/1933 (H1N1) virus is considered to be a neurotropic virus and therefore we tested the presence of the virus also in the brains of mice. The aim was to determine whether the neurotropism of this virus could be influenced by the introduction of described HA2 mutations. Although we did not detect the infectious virus in mouse brain in any of the experimental groups, the presence of vRNA was confirmed (Fig. 6). We therefore analysed the amount of vRNA in brains by quantitative real-time PCR. The vRNA in the brains of mice infected with wt virus was detected on the second day post infection in a low amount and with a slight increase on the 8 days p.i. On the other hand, vRNA in brains of mice infected with mutant 64 was present throughout the whole time course monitored, i.e. 10 days, and on days 2–6 post-infection at a more than 1000-times higher amount than in wt-infected mice. The reason for this extensive vRNA expression will be studied in the future. In brains of mice infected with mutant virus D, vRNA was detected from the 4th to the 10th day p.i., but it was not present in all tested mice. Interestingly, we were able to detect vRNA in the brain of mice infected with mutant 66 only at late time points post-infection, particularly from day 6 to 10.

## Discussion

The replication efficiency, virulence, pathogenicity and transmissibility of influenza viruses depend on a number of viral and host factors [2, 4, 12, 13, 23–25]. HA, the main surface gp, is considered to be a key viral factor. It has essential functions in influenza virus replication: it is responsible for binding to the host cell receptor and for the fusion of viral and endosomal membranes. To mediate the membrane fusion, HA undergoes conformational changes which are pH-dependent. Several previous studies have focused on the pH-dependent conformational changes in HA [6, 10, 26–31]. It was shown that the HA conformation is influenced by ionizable amino acids in several important regions of the HA trimer, including the fusion peptide, the coiled-coil region of the light chain of HA, and the area of interaction between the HA1 and HA2 subunits [4, 32]. At low pH, the region of amino acids 56–75 of HA2 gp undergoes the transition from loop to helix, which is an essential step for exposure of the N terminus of HA2 out from the HA trimer needed for its insertion into the target membrane [3, 8, 33, 34]. The pH required for HA refolding and fusion activation varies among different strains and is closely related to the pH optimum of fusion and subsequently influences the replication ability of the virus. Therefore, the pH optimum of fusion is an important factor affecting virus infectivity and virus adaptation to a new host [32].

To determine the impact of changes in the fusion pH optimum on the biological properties of IAV, we prepared mutants with amino acid substitutions in the area of interaction between the HA1 and HA2 subunits. The basic amino acid histidine, the charge of which is extremely pH-sensitive under physiological conditions, was chosen in our study as the most suitable substituent to generate an HA trimer mutant with altered pH stability. Histidine was introduced into the HA2 gp of wt virus A/WSN/33(H1N1), namely at position 64 instead of threonine (mutant virus 64) and at position 66 (mutant virus 66) instead of valine. Moreover, a double HA2 mutant comprising both substitutions, T64_2_H and V66_2_H, was prepared.

We examined how these substitutions influence the pH optimum of fusion and the *in vitro* as well as *in vivo* properties of A/WSN/33 (H1N1) influenza viruses. First, a luciferase method was used to monitor the pH optimum of viral and cell membrane fusion mediated by particular mutants. The maximum fusion activity of mutant 64 was at pH 5.4-5.6, which was comparable with that of the wt virus. Mutant viruses 66 and D had a lower pH maximum of fusion, estimated at pH 5.1-5.3. By monitoring IAV fusion activity in the range from neutral pH towards low pH, we showed that a pH of 6.0–5.8 was a threshold pH at which fusion activity became detectable. These results are consistent with published data [35, 36].

For *in vitro* analysis of replication of mutant viruses we chose three different cell lines – MDBK, A549 and VERO. It is generally known that MDBK cells promote effective virus replication, which we confirmed experimentally. In contrast, the lowest virus titres were obtained by propagation of viruses in human lung cell line A549. The *in vitro* replication profile of the mutant 64 was the most similar to the wt virus. In all cell lines, lower replication of 66 and D mutants in comparison to wt and 64 viruses was observed, but only in the A549 cell line were differences in growth rates of 66 and D mutants vs. wt and 64 viruses significant (*P*<0.01).

The difference in the replication ability of virus mutants *in vitro* (but also *in vivo*) is determined by many factors. One of them is the stability of virus infectivity after exposure to low pH. The influence of HA pH stability on virus infectivity was analysed, statistically evaluated and the 50 % reduction of virus infectivity was determined. This analysis showed that the D mutant was most sensitive to pH decrease, where 50 % of infectivity was observed between pH 6.2– and 6.0, while other viruses dropped to 50 % at lower values, between pH 6 and 5.8. The highest low pH sensitivity of the D mutant could be attributed to two histidine residues in this area, present at positions 64 and 66 of HA2 gp instead of theronin and valin. At lower pH (from 6 to 5) the infectivity of all viruses rapidly decreased. However, the low pH pre-exposure influences not only the conformation of HA. The impact of pre-exposure of virus to low pH is more complex and can have many consequences, as its impact on the NA or M2 ion channel activity, which in cooperation with HA and host factors also influences ribonucleoprotein release from the viral particle, resulting in the character of virus infectivity [37].

Similar to *in vitro* replication activity, the *in vivo* replication of mutants 66 and D was reduced in comparison with the wt and mutant 64. A 30-fold higher amount of the 66 mutant virus and a seven-fold higher amount of the D mutant than of wt and 64 viruses was required to achieve the 1 LD50 infection dose, i.e. 50 % lethality in mice. In accordance, inspection of the lung damage of mice infected with 1 LD_50_ of the examined virus unambiguously pointed to a milder course of infection in mice infected with mutant virus 66 than with wt or 64 viruses. In mutant virus 66, however, we were able to detect vRNA in mouse lungs also at later points and, surprisingly, we detected RNA even on day 15 p.i. (in two of three mice, data not shown). Nevertheless, this mutant as well as mutant D, which showed lower replication activity *in vitro* and required several times higher dose of virus to achieve 50 % lethality in mice, reached the comparable virus titres in mouse lungs as did the wt and mutant virus 64. However, 66 and D mutants were eliminated from mouse lungs more slowly than the wt and 64 virus. Namely mutant D was detected in mouse lungs even on day 10 p.i.

*In vivo*, the T64H substitution in mutant 64 caused greater lung damage in infected mice (70–90 %), which was comparable to the wt virus, while the V66H substitution confer to virus 66 a milder pathogenicity, resulting in 10–20 % lung damage. The increased manifestation of lung damage in D mutant-infected mice compared to 66 virus could be attributed to the second, T64H substitution introduced into the D mutant.

We hypothesized that the higher amount of virus is needed for the lethal dose 1 LD_50_, the longer time for its elimination is needed. Similar results were described by Fislová *et al*. [21], where three viral strains of different virulence, the low virulent A/Dunedin/4/73 (H3N2), medium virulent A/Mississippi/1/85 (H3N2) and highly virulent A/PR8/34 (H1N1) viruses were compared.

As the influenza A/WSN/33 (H1N1) virus strain used in this study is considered to be neurotropic, we attempted to detect infectious virus not only in the lungs but also in the brains of infected mice. There are several theories explaining how the neurotropic IAV enters the brain. One possibility is that the virus enters the central nervous system via the olfactory nerve (*Nervus olfactorius*), as previously experimentally demonstrated in mice [1, 38–41]. The olfactory nerve begins in the nasal cavity, and therefore, after intranasal infection, the virus can spread along the axons to the olfactory bulb (*Bulbus olfactorius*) [39, 40, 42]. Here the virus may persist for some period of time without its further propagation due to control by the mechanisms of innate immunity (cytokines and CD8+ cells) of the host [39, 42]. In our experiments we did not detect infectious virus in brain, but showed the presence of vRNA of all examined viruses in mouse brains. However, the relative amount of expressed vRNA in mouse brains at particular time points after the infection differed among the examined viruses. We observed that vRNA in brains of wt- and 64-infected mice was expressed already on the second day after infection, but vRNA of the 64 mutant was expressed at much higher levels than that of the wt. Such a difference in expression level of wt and 64 vRNA in mouse brains probably results from different tissue tropism of wt and 64 viruses caused by mutation in HA (T64H) in relation to neuronal cells. Changes in the receptor binding activity of IAV caused by mutation in HA2 gp have been reported by other authors [30]. However, transport of IAV via the central nervous system is a very complex process, and can be influenced also by other factors which have yet to be clarified [39]. On the other hand, vRNA of the 66 and D mutants was detectable in brains only at later points post-infection, but remained until the end of the monitored time interval. This can be linked to the lower virulence of 66 and D mutants in comparison to the wt and probably to the milder induction of immune defence machinery as those induced by the wt virus [43].

The mutation introduced into HA2 gp had an impact on the pH optimum of fusion in the case of mutant virus 66 or D, where the broad pH maxima were shifted to lower values. There was recorded a difference in sensitivity of mutant 66 and D infectivity to low pH when compared to the wt. The changes are very subtle, so it is hard to say if this is in accordance with previous observations, namely that increased stability of IAV at low pH corresponds to decreased fusion pH, while a decreased HA stability corresponds to higher fusion pH [44]. However, there is the question of A/WSN/33 (H1N1) viral neuraminidase, which is unique among IAVs and it can skew our data.

However, it should be stressed that although changes in *in vitro* virus replication and virus pathogenicity were detectable also in the 64 mutant virus that showed no shift in the pH optimum of fusion, none of the introduced mutations resulted in increased virulence and the worsening of the course of infection *in vivo*. The differences in *in vitro* and *in vivo* replication of mutant viruses in comparison to the wt were accompanied also by other changes such as lung damage, the persistence of infectious virus in lungs and vRNA in brains of infected mice. After infection of mice with an equal lethal dose of virus, we observed that the more virulent was the virus used for infection, the earlier its titre in lungs decreased.

In summary, the constructed IAV mutants 66 and D had lowered virulence in comparison to the wt. Both of them (mutant 66 and D) had broad maximum of fusion activity shifted to lower pH in comparison to the pH of fusion maximum of wt or 64 mutant virus. Mutants 66 and D were eliminated from lungs of infected mice later than the wt. However, vRNA of mutant 64 with fusion optimum in the same pH range as the wt, and with similar *in vitro* and *in vivo* characteristics as the wt, was present in brains of mice in a higher amount in comparison to wt-infected mice and therefore could be more neurotropic than the wt. Previous data show that fusion pH optimum of highly pathogenic avian IAV, in particular isolated from poultry, is higher than that of human IAV. This parameter, linked to the pH at which virus HA is stable, can influence IAV tropism, which plays an important role during interspecies transmission of IAVs and in IAV ecology [5, 7].

In general, HA activation pH values for IAV typically range from 4.8 to 6.2 [5]. However, they differ among IAVs depending on host origin of the isolate. Swine IAV isolates have higher activation pH (5.6–5.7) than human pH1N1 isolates from 2009 (5.5–5.6) or isolates from 2010 to 2012 (5.2–5.4) [5]. From published data it appears that newly emerged IAV isolates have often less stable HA than those that are adapted to humans. In our experiments we obtained mutants with pH stability similar to human pH1N1 isolates from 2009 (66 or D mutant), as well as mutant 64 and wt, the pH stability of which was comparable to older IAVs (pH 5.2–5.4). All our viruses are more stable than swine viruses. Using a reverse genetic system for construction of the mutants analysed here, we eliminated all other variables from our observations, and also the properties of NA and M proteins (M1 and M2), which also influence the pH stability of IAV, and their phenotypic properties. Therefore, the prediction of destabilizing mutations in HA, with an impact on the replication ability of IAV in a given host, needs to be experimentally verified. The pH stability of HA is an important factor influencing the immunogenicity of vaccines composed of HA subunits or inactivated vaccine and must be taken into the account for the development of new vaccines [45].

It cannot be excluded that some other, undescribed HA2 fusion mutants could emerge in nature, namely of avian or swine IAV, with replication ability in a new mammalian/human host, but with a more virulent and more pathogenic phenotype, and thus representing a threat to humans [5].
